# Transgenerational effects of innate immune activation in broiler breeders on growth performance and immune responsiveness

**DOI:** 10.1016/j.psj.2021.101413

**Published:** 2021-07-30

**Authors:** Michel B. Verwoolde, Jürgen van Baal, Christine A. Jansen, Elisabeth A.M. Graat, David M. Lamot, Aart Lammers, Lieske van Eck

**Affiliations:** ⁎ Adaptation Physiology Group, Department of Animal Sciences, Wageningen University and Research, Wageningen P.O. Box 338, 6700 AH, the Netherlands; † Animal Nutrition Group, Department of Animal Sciences, Wageningen University and Research, Wageningen P.O. Box 338, 6700 AH, the Netherlands; ‡ Department of Biomolecular Health Sciences, Division of Infectious Diseases and Immunology, Faculty of Veterinary Medicine, Utrecht University, 3584 CL Utrecht, the Netherlands; § Cell Biology and Immunology Group, Department of Animal Sciences, Wageningen University and Research, Wageningen P.O. Box 338, 6700 AH, the Netherlands; # Cargill Animal Nutrition Innovation Center, Veilingweg 23 5334 LD, Velddriel, the Netherlands

**Keywords:** chicken, transgenerational, β-glucan, lipopolysaccharide, innate immunity

## Abstract

The impact of transgenerational effects on growth performance and immunity has not yet been studied extensively within the poultry husbandry sector. An important factor is the impact of the hens on the physical well-being and fitness to the environment of the offspring. This study is the first to investigate the effect of stimulating the maternal innate immune system with lipopolysaccharides (**LPS**) or β-glucan on growth performance and immune responses in the next generation. Transgenerational effects and consequences of these maternal treatments were further examined using a necrotic enteritis (**NE**) challenge model in the offspring. We show that offspring of LPS-treated broiler breeders have a higher feed efficiency from 14 to 21 days of age, that is, the period just after the NE challenge. Moreover, more broiler chickens with intestinal lesions after the NE challenge were found in the offspring of the LPS-treated broiler breeders. Both the LPS and β-glucan maternal treatments resulted in transgenerational effects on blood-derived monocytes by showing a tendency of decreased IL1β mRNA levels after ex vivo LPS stimulation. These data are a first indication that broiler breeder hens can affect immune responsiveness and feeding efficiency of their offspring in a transgenerational manner.

## INTRODUCTION

Within the broiler husbandry sector, the containment of infectious diseases has always been a significant focus area. An important factor is the impact of the hens on the well-being and fitness of their offspring. It has been hypothesized that the hens prepares her offspring for their environment through transmission of maternal antibodies (Gluckman et al., 2007; Hasselquist and Nilsson, 2009). When the living environments of hens and her offspring are equal, it is supposed that this next generation is better prepared. However, no research has been done yet on investigating the mode of action and potential benefits of these transgenerational effects, and within this, the role of the innate immune system.

Invertebrates and plants lack the presence of an adaptive immune system but have the ability to develop protection against pathogens in a transgenerational manner (Slaughter et al., 2012; Rosengaus et al., 2017), implying a memory function of the innate immune system. Because of this memory function in invertebrates, it has been proposed that the innate immune system of vertebrates also has a memory function, which contributes to this protection (Netea et al., 2011; Netea et al., 2015). Activation of the innate immune system in mammals resulting in enhanced responsiveness to subsequent triggers leading to polyspecific resistance, that is, innate immune memory has been termed trained innate immunity (Netea et al., 2020). Trained innate immunity in mammals involves epigenetic changes which have been found to have long-term effects (Kleinnijenhuis et al., 2014). Therefore, it has been suggested that these epigenetic effects could also affect the innate immune system of chickens in the next generation (Berghof et al., 2013), comparable to plants, invertebrates, and mammals. Since broiler chickens have a short lifespan relatively to laying hens, these epigenetic effects on innate immune cells would therefore especially be more relevant for broiler chicks, knowing that the adaptive immune functions in chickens have not yet fully developed during the first weeks of their short lives (Bar-Shira et al., 2003; Lammers et al., 2010).

In previous studies, we demonstrated trained innate immunity in poultry through ex vivo experiments with blood-derived primary monocytes (Verwoolde et al., 2020a,b). It is unknown, however, if trained innate immunity can influence the offspring's physiology. Therefore, the aim of the present study is to investigate if stimulation of the maternal innate immune system with microbial-associated molecular patterns (**MAMPs**) has an effect on the offspring's growth performance parameters and immune responsiveness.

We have immunized broiler breeders with 2 innate immune-stimulating components, the MAMPs lipopolysaccharide (**LPS**) or β-glucan to evoke possible transgenerational effects. These components act via Toll-like receptor 4 and Dectin-1 receptor signaling, respectively, which are expressed on innate immune cells including monocytes (Dil and Qureshi, 2002; Brown et al., 2003; Nerren and Kogut, 2009; Goodridge et al., 2011). Although an intensive BLAST search in the *Gallus gallus* genome database (GRCg6a: build GCF_000002315.6) did not result in the identification of a dectin-1 chicken homologue, a dectin-l like β-glucan receptor is likely to be present on chicken heterophils and PBMCs (peripheral blood mononuclear cells), which have been found to respond to the dectin-1-specific agonist curdlan by an oxidative burst (Nerren and Kogut, 2009). The transgenerational effects and consequences of these maternal treatments on growth performance and immune response parameters of their offspring were examined using a necrotic enteritis (**NE**) challenge model (Kocher et al., 2004; Mikkelsen et al., 2009; Lensing et al., 2010; Wu et al., 2010; Rodgers et al., 2015; Gharib-Naseri et al., 2021). Effects of immunological stress caused by pathogens are generally reflected in decreased growth performance parameters (Lovland and Kaldhusdal, 2001). Differences on immune responsiveness and growth performance parameters are therefore considered as a good measurement to identify transgenerational effects of the maternal treatments.

This study should be considered as an initial step in identifying potential transgenerational effects of innate immune activation in poultry.

## MATERIALS AND METHODS

### Animals and Ethical Statement

This study was approved by the Animal Welfare Committee of Wageningen University and Research in accordance with Dutch laws and regulations on the execution of animal experiments (no: AVD1040020185427).

### Experimental Design

**Figure 1:**
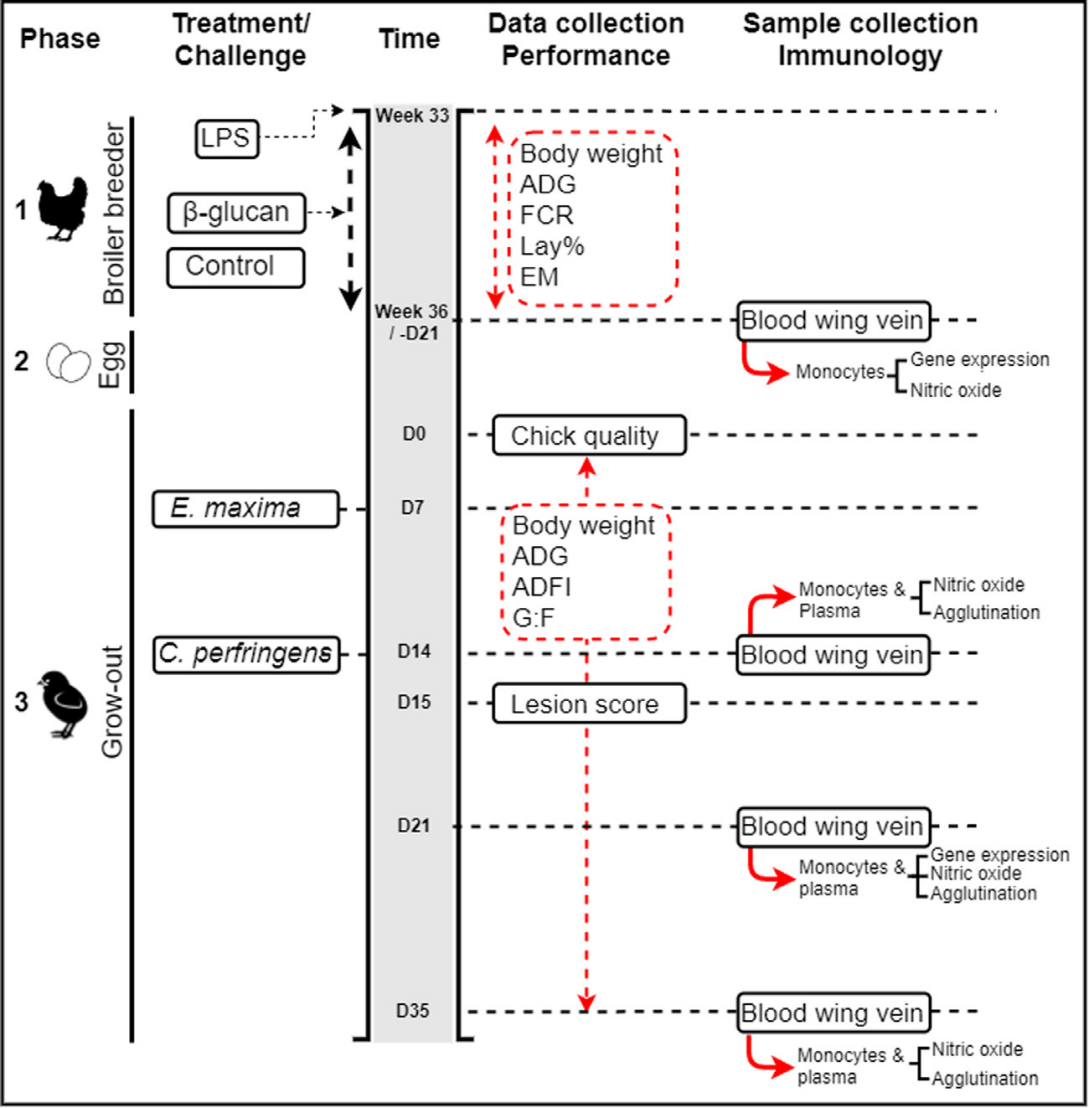
Timeline of the transgenerational experiment. The experiment was divided into three phases: broiler breeder (1), egg incubation (2), and grow-out (3). Treatments, challenges, data collection and sample collection are shown. Time indicates the moment when the different handlings were done. Data collection for performance purposes was done during the entire experiment and indicated by the red-dashed arrows. Measurement of the Chick quality (Tona score) and intestinal lesions (Lesion score) was done ones, respectively at d 1 and d 15. The *E. maxima* (d 7) and *C. perfringens* (d 14) challenge are part of the necrotic enteritis challenge. Abbreviations: ADFI, average daily feed intake; ADG, average daily gain; EM, egg mass; FCR, feed conversion ratio; G:F, gain to feed ratio; Lay%, laying percentage .

Transgenerational effects of maternal stimulation of the innate immune system were studied by treatment of broiler breeder females with either LPS or β-glucan. The effects on the offspring performance were evaluated by exposing them to an NE challenge (Figure 1). A schematic design of the experiment is shown in Figure 1. The experiment was divided into 3 phases. In phase 1, the broiler breeder phase, a randomized complete block design was applied with 3 treatments (control, LPS, or β-glucan) and 162 hens per treatment, with 6 replicates per treatment. In phase 2, the egg incubation phase, a total of 90 eggs of each broiler breeder treatment group were collected, incubated, hatched, and day-old chick quality was studied. In phase 3, the grow-out phase, 90 male chicks of each of the 3 broiler breeder treatment groups were raised until 36 d of age and challenged with necrotic enteritis.

### Animals, Housing, and Management

#### Broiler Breeder Phase (1)

A total of 486 female and 54 male Ross 308 broiler breeders at 20 wk of age (Pluvita B.B., Apeldoorn, the Netherlands), derived from the same grandparent flock, were allocated to 18 floor pens with 27 hens and 3 male chickens per pen at the Cargill Animal Nutrition Innovation Center (Velddriel, the Netherlands). The pens were divided over 6 blocks with 3 pens per block and hens were allocated to pens based on body weight, to create blocks with similar body weights and low variation within block. Treatments were randomly allocated within weight blocks. The hens were housed in floor pens of 6.24 m^2^ with deep littered floor (flax) covering 1/3rd of the pen and elevated floor with plastic slats covering 2/3rd of the pen. Per pen, 240 cm of nest box was available and water was provided for 6 h per day according the broiler breeder guidelines. Females were fed with feeding bowls and males were fed in a separate feeding trough. Diet composition and feeding regime was based on the broiler breeder guidelines (Aviagen-EPI, 2017) (see Supplementary material). Daily feed allowance was calculated weekly per pen, based on average pen body weights and egg production. Temperature was maintained at 20°C by floor heating. Photostimulation started at 22 wk of age with 10 h of light (20 lx), after which day length was gradually increased with 1 h and 10 lx per wk to 12L:12D.

#### Egg Incubation Phase (2)

At 35 wk of age, a total of 15 first class eggs per pen were randomly collected for 6 consecutive days for incubation resulting in 15 × 6 × 6 = 540 eggs per treatment. Eggs were incubated according to a standardized protocol (see Supplementary material).

#### Grow-Out Phase (3)

For the grow-out phase, the newly hatched chicks were feather sexed and 90 healthy male broiler chickens per broiler breeder treatment were randomly selected and allocated to 18 pens in a grow-out facility of the Cargill Animal Nutrition Innovation Center (Velddriel, the Netherlands). Per pen 15 male broiler chickens were housed that originated from the same broiler breeder pen. The 3 broiler breeder treatments were randomly allocated within 6 blocks, resulting in 6 replicates per treatment. Pens had a raised floor covered with wood shavings. Artificial lighting was set for 23 h per day from d 0 to d 2, 20 h per day from d 2 to d 6 and 18 h per day from d 7 to d 34. The temperature was set to gradually decrease by 0.5°C per day during the first 14 d, starting at 34°C on d 0. From d 14 onward, the temperature was set to decrease gradually to a final temperature of 20°C on d 36. Each pen was equipped with cup drinkers adjustable in height. During the first 3 wk, feed was supplied using a tower feeder placed in the pen. From d 21 onward, the feed was supplied in a metal feeder trough placed in front of the pen. All treatments received the same diets and feeding regime according to the breeder guidelines (see Supplementary material). On d 3 and d 21, all broiler chickens were vaccinated against ND by means of an intramuscular injection.

### Experimental Treatments

#### Broiler Breeder Phase (1): Innate Stimulation With MAMPs

At 33 wk of age, broiler breeders received 1 of the 3 treatments (Control, LPS, or β-glucan). Broiler breeder hens of the LPS group were intratracheally inoculated using a blunted needle with 1 mg/kg BW LPS from *Escherichia coli* serotype O55:B5 (L2880, Sigma-Aldrich corporations, St. Louis, MO) dissolved in 0.5 mL PBS (Gibco, Life Technologies Ltd., UK). Broiler breeder hens of the control and β-glucan treatment groups were inoculated intratracheally with only the solvent 0.5 mL PBS (Gibco, Life Technologies Ltd.). Broiler breeder hens of both the control group and the LPS group were fed a control diet, without β-glucan, formulated according to the breeder guidelines (Aviagen-EPI, 2017). Broiler breeder hens of the β-glucan treatment group received a similar diet, in which 0.05% of corn was exchanged for the β-glucan feed additive (Macrogard, Orffa, Werkendam, the Netherlands) (Table S2).

#### Grow-Out Phase (3): NE Challenge

All broiler chickens were challenged using an NE model based on Lensing et al. (2010). The challenge was performed in two steps. First, a mild *Eimeria maxima* infection was used to enable colonization with *Clostridium perfringens* in the second step. All broiler chickens were orally inoculated on d 7 with 1 mL of *E. maxima* (4,500 sporulated oocysts per mL; Weybridge strain; Royal GD, Deventer, the Netherlands). All feed was removed 2 h prior to inoculation. After a 7-d incubation time for coccidiosis to develop, all chicks were orally inoculated on d 14 with 1 mL of a pathogenic strain of *Clostridium perfringens* (code GD 5.11.53; 10^8^ CFU/mL liver broth; Royal GD) to induce the necrotic enteritis. All feed was removed 2 h prior to inoculation.

### Performance Data Collection

#### Broiler Breeder Phase (1)

Broiler breeder hens were weighed individually at 33, 34, 35, and 36 wk of age. Based on weekly body weights, ADG was calculated and feed was provided restrictedly and recorded daily. Eggs were collected twice a day and numbers of eggs were recorded daily per pen to calculate lay percentage per week (number of eggs per week divided by number of hens). One day a week, all eggs collected the previous day were weighed per pen and average egg weight was calculated. Based on egg weight and lay percentage, egg mass was calculated (egg weight multiplied by lay percentage, g). The feed conversion ratio for egg mass (**FCR**; kg of egg mass per kg of feed consumed) was calculated using calculated egg mass and ADFI. Mortality was recorded daily. Two hens per pen were randomly chosen at the start of the study and were color marked on the neck or back or both for individual identification to enable blood sampling. Blood samples were taken at 33 and 36 wk of age. Per blood sampling 3 mL heparinized blood was collected and immediately transported to the laboratory for isolation of leucocytes. All hens were fed 2 h prior to sampling.

#### Egg Incubation Phase (2)

Prior to incubation, all eggs were weighed. Eggs were candled on d 10 and d 17 of incubation and empty eggs or dead embryos were recorded and removed. After hatch, total number of dead chickens, live pipped eggs and hatched eggs were recorded. Fertility (ratio of filled eggs, including embryonic mortality, of total), mortality (ratio of early and late mortality of fertile eggs) and hatchability (ratio of hatched chicks of total) were calculated. After hatch, 2 female chicks per experimental unit were randomly selected and chick quality was assessed using part of the score as described by Tona et al. (2003) (see Supplementary material). Subsequently, the same chicks were weighed, euthanized, and dissected to measure residual yolk weight and calculate yolk-free body mass (**YFBM**). Yolk weight as percentage of live body weight was calculated.

#### Grow-Out Phase (3)

On d 0 group BW was determined and averaged per pen. Individual body weights were determined on d 7, d 14, d 21, and d 35. Feed consumption was recorded per pen on the same days. The gain to feed ratio (**G:F**; kg of weight gain per kg of feed consumed) was calculated based on calculated ADG and ADFI. Mortality was recorded daily. At the start of the trial, 5 broiler chickens per pen were randomly selected and marked for blood collection on d 14 (2.75 mL per broiler chicken), d 21 (3 mL per broiler chicken) and d 36 (5 mL per broiler chicken). Heparinized blood samples were taken from the wing vein before *C. perfringens* inoculation on d 14 and before diet changes on d 14 and d 21. The blood samples were transported to the laboratory for isolation of leucocytes within 2 h under controlled conditions (see below). On d 15, one day after *C. perfringens* inoculation, two broiler chickens per pen (excluding the marked broiler chickens) were randomly selected, and consequently weighed and euthanized. Lesion scoring was performed according standardized procedures (Lensing et al., 2010). First, the broiler chickens were, through a blind experimental approach, visually scored for *E. maxima* (characteristic lesions: hemorrhages, blood, and orange mucus) and *C. perfringens* (characteristic lesions: white, brown, and/or gray spots). The *E. maxima* was scored from 1 to 4 in the second loop of the duodenum and jejunum till the Meckel's diverticulum (score 1 = one or some hemorrhages; score 2 = several hemorrhages; score 3 = many hemorrhages, orange mucus in the lumen; score 4 = many hemorrhages, mucosal damage, free blood in the lumen). The *C. perfringens* was also scored from 1 to 4 in the gizzard towards Meckel's diverticulum (score 1 = one to five lesion present in the intestine; score 2 = more than 5 single distinguished lesions in the intestine; score 3 = lesions merge or extend to a surface of more than 1 cm^2^; score 4 = pseudo membranes present in the intestine causing death).

### Immunological Data Collection

#### Isolation, Culture, and Stimulation of Primary Monocytes

As described above, heparinized blood from broiler breeder hens and broiler chickens was collected at several timepoints during the experiment (see Figure 1 for timeline). Primary monocytes from blood were isolated as described previously (Verwoolde et al., 2020b). Briefly, mononuclear cells were purified using histopaque-1119 (density: 1.119 g/mL, Sigma-Aldrich corporations) followed by density gradient centrifugation (700 × *g*, 40 min at room temperature) and seeded at a concentration of 1 × 10^6^ cells per well in a 96-well flat bottom plate (CELLSTAR, Greiner Bio-One, Alphen aan den Rijn, the Netherlands), followed by incubation at 41°C in 5% CO_2_ and 95% humidity overnight. The next day, nonadherent cells were washed away with prewarmed (41°C) complete cell culture medium (i.e., RPMI 1640 supplemented with 25 mM HEPES, Glutamax, 10% heat-inactivated chicken serum and 50 U/mL penicillin and 50 μg/mL streptomycin; all from Gibco, Life Technologies Ltd.).

Cells were collected for RT-qPCR analysis after a 24 h stimulation with 200 μL complete culture medium (control) or 200 μL LPS from *Escherichia coli* serotype O55:B5 (f.c. 10 µg/mL, L2880, Sigma-Aldrich corporations). The cells were washed with ice-cold PBS, directly lysed with RLT lysis buffer and stored at −80°C until further RT-qPCR analysis (Qiagen, Hilden, Germany). From an identical, in parallel-performed experiment, cell culture supernatant was collected after 48 h for subsequent analysis of nitric oxide (**NO**) production. For NO production assays, cells were stimulated with LPS (1 or 10 µg/mL, or LPS (10 µg/mL) + IFNγ (0.1 µg/µL) in complete culture medium. Blood plasma collected after the density gradient centrifugation was stored at −20°C until the agglutination assay.

#### NO Production

Accumulated NO in the medium upon stimulation was indirectly measured by quantifying nitrite (NO_2_^−^) concentration in the culture medium, using the Griess reaction assay as previously described (Verwoolde et al., 2020a). Briefly, medium was collected from stimulated macrophages and combined with Griess reagent in a 1:1 ratio. The NO_2_^−^ concentration was determined by measuring the optical density at 540 nm with a spectrophotometer (Multiscan, Thermo Fisher Scientific, Waltham, MA). The results were interpolated on a standard curve made by serially diluting a sodium nitrite solution (NaNO_2_) in the range from 0 to 100 μM.

Cell viability was assessed with an Alamar Blue (**AB**) assay (see Supplementary material). This assay allowed the establishment of the relative variation in the number of viable cells in the wells of the 96-wells plates, because only viable cells will reduce the blue colored resazurin into the red colored resorufin. Reduction of the AB solution was quantified with a spectrophotometer. The amount of reduced resazurin, which reflects the number of viable cells, was used to normalize the corresponding NO production for the same well to assess the amount of NO per viable cell.

#### Total RNA Isolation and qPCR Analysis

Total RNA isolation and qPCR procedure are described in more detail elsewhere (Verwoolde et al., 2020a). Briefly, total RNA was isolated and subsequently subjected to a DNase digestion treatment. RNA quantity and purity were measured with a NanoDrop 1000 Spectrophotometer (NanoDrop Technologies LCC, Thermo Fisher, Wilmington, DE). RNA quality was determined using the Agilent 2100 Bioanalyzer according manufacturer's instructions (Agilent Technologies, Santa Clara, CA). Total RNA (50 ng) was reverse transcribed into complementary DNA (**cDNA**) using random hexamer primers (Roche Diagnostics, the Netherlands) and the SuperScript III Reverse Transcriptase kit (18080044; Invitrogen, Breda, the Netherlands). The qPCR assay was based on a 20 µL volume design using the SensiFAST SYBR Lo-ROX Kit (Bioline, Meridian Bioscience Inc., Cincinnati, OH) together with a 5 µM specific sense and antisense primer set (Table 1), and was performed with a QuantStudio 5 Real-Time PCR system (Applied Biosystems, Thermo Fisher Scientific Corporation, Foster City, CA). Amplification conditions were 95°C for 2 min, followed by 40 cycles of 95°C for 5 s and 60°C for 20 s each. Melting curve analysis confirmed specific amplification of a single PCR product. The results were interpolated on a standard curve made by 10× serial dilution of a known amount of corresponding cDNA product of the target gene. Absolute mRNA quantities were normalized to the geometric mean of three internal reference genes (Table 1), which were identified as being the most stable genes as determined with Normfinder algorithm software (Andersen et al., 2004).

**Table 1:** Primers used for RT-qPCR.

Target^1^	Sequence^2^	Accession no.
Internal reference genes		
ACTB	F: 5’-GCCCTGGCACCTAGCACAAT-3’	NM_205518
	R: 5’-GCGGTGGACAATGGAGGGT-3’	
IPO8	F: 5’-ACCTCCGAGCTAGATCCTGT-3’	XM_015287054
	R: 5’-GGCTCTTCTTCGCCAACTCT-3’	
GAPDH	F: 5’-ATCCCTGAGCTGAATGGGAAG-3’	NM_204305
	R: 5’-AGCAGCCTTCACTACCCTCT-3’	
Genes associated with inflammation		
IL-1β	F: 5’-GACATCTTCGACATCAACCAG-3’	XM_015297469
	R: 5’-CCGCTCATCACACACGACAT-3’	
iNOS	F: 5’-CTACCAGGTGGATGCATGGAA-3’	NM_204961
	R: 5’-ATGACGCCAAGAGTACAGCC-3’	

IL-1β and iNOS were used to measure an effect of maternal treatments on proinflammatory immunity.

1 ACTB, actin beta; GAPDH, glyceraldehyde-3-phosphate dehydrogenase; IL, interleukin; IPO8, Importin 8; iNOS, inducible nitric oxide synthetase.

2 F, forward; R, Reverse.

#### Agglutination Assay

An agglutination assay was used to determine the relative amount of agglutinating antibodies against *Clostridium perfringens type A necrotic enteritis toxin B-like* strain in blood plasma. An inactivated bacterial suspension of this strain with a concentration of 10^9^ bacteria/mL was commercially provided (Royal GD). The suspension was diluted to reach an optimal concentration with optical density of 0.450 (cuvette dimension of 10 × 10 mm) using a 510-nm filter (Evolution 201 UV–Visible, Thermo Scientific, Waltham, MA). Next, a volume of 25 µL of each plasma sample was two-fold serial diluted in PBS (Gibco, Life Technologies Ltd., UK) in a 96-well round bottom plate (CELLSTAR, Greiner Bio-One). Every row represented one sample and the last column was used as negative control. Next, 25 µL of the optimized inactivated bacterial suspension was pipetted into the wells and suspensions were mixed for 10 s using a microplate shaker, followed by a 12-h incubation period at 4°C. Antibody titers were scored after placing the plates at a 45° angle for 30 s in order to enhance visualization of agglutination. The antibody titer represents the number of the last column where agglutination was still present.

### Statistical Analysis

#### Performance Data

Data were analyzed using pen as the experimental unit. Model assumptions, that is, normality and equal variance of the error terms, were checked by inspection of the residual plots. Data were subjected to mixed model analyses, using R Studio (R Studio version 1.1 2009-2018; RStudio, Inc., Boston, MA). For the broiler breeder phase and the grow-out phase, the following statistical model was used:

$$Y_{ij}=\mu +\alpha_{i}+B_{j}+\varepsilon_{ij}$$

where Y_ij_ = dependent variable, μ = overall mean, α_i_ = fixed effect of treatment (i = control, LPS or β-glucan), Bj = random block effect (j = 1 - 6) and εij = residual error.

For the egg incubation phase, the following statistical model was used:

$$Y_{ijk}=\mu +\alpha_{i}+B_{j}+\alpha {\left(C\right)}_{ijk}+\varepsilon_{ijk}$$

where Y_ij_ = dependent variable, μ = overall mean, α_i_ = fixed effect of treatment (i = control, LPS or β-glucan), B_j_ = random block effect (j= 1 - 6), α(C)_ijk_ = random nested effect of breeder pen within treatment effect (k = 1-24) and ε_ijk_ = residual error.

Nonbinomial data are expressed as least square (**LS**) means. LSmeans were compared after being corrected with a Tukey test for multiple comparisons and effects were considered to be significant when *P* ≤ 0.05. *E. maxima, C. perfringens*, mortality, fertility, hatchability, and Tona score data were analyzed as binomial distributed data, using the same statistical model. Tona score data was non-normally distributed and normalized using a Box-Cox transformation (Box and Cox, 1964). Effects were considered to be significant when *P* ≤ 0.05.

#### Immunological Data

Immunological data were analyzed with SAS statistical software (SAS 9.4, SAS Institute Inc., Cary, NC). The same statistical approach was used for the broiler breeder phase (1) and the grow-out phase (3) with the following statistical model:

$$Y_{ij}=\mu +\alpha_{i}+B_{j}+\varepsilon_{ij}$$

where Y_ij_ = dependent variable, μ = overall mean, α_i_ = fixed effect of treatment (i = control, LPS or β-glucan), B_j_ = ex vivo LPS stimulation (mRNA: j= 1 - 2; NO: j= 1 – 4; Agglutination: n/a) and ε_ij_ = residual error.

Mixed models were created with relative mRNA expression, nitric oxide and agglutination titer as dependent variables. Since pen as random effect was very small and nonsignificant, an ordinary linear regression model was performed. Model residuals were assessed for normality by creation of histograms and Q-Q plots. The used explanatory variable was maternal treatment. For the response variables nitric oxide and agglutination titer, the variable age was included in the model as well. Furthermore, interaction effects were tested for significance. Effect of maternal treatment and treatment in the offspring on relative mRNA expression was tested within each gene, being iNOS and IL1-β. Immunological data are expressed as means and effects were considered to be significant when *P* ≤ 0.05.

## RESULTS

### Effects of Immunization With Innate Immune-Stimulating Components on Performance and Immune Responsiveness in Broiler Breeders, Eggs, and on Chick Quality

**Figure 2:**
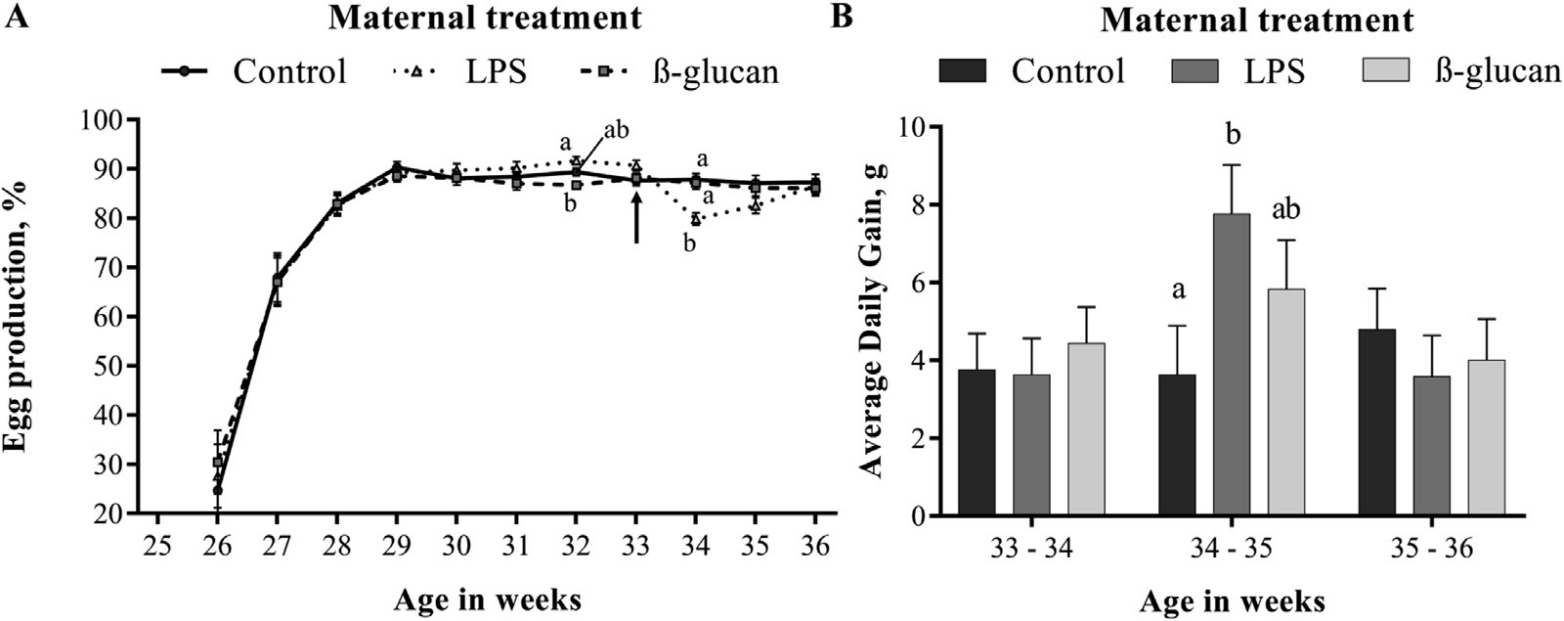
Maternal treatment effect on egg production and ADG during the broiler breeder phase (1). LPS was inoculated at the beginning of wk 33 (arrow) and simultaneously the treatment with β-glucan was also started at this timepoint. (A) Egg production in wk 34 for broiler breeder hens that were inoculated with LPS showed lower egg production (∼8% compared to control). (B) ADG in wk 34 to 35 showed that growth was significantly higher in the LPS treatment group compared to the control. (A and B) N = 6 pens per treatment with 27 hens per pen. Data is represented as lsmeans. Effects are represented as superscripts and were considered to be significant when *P* ≤ 0.05. Abbreviations: ADG, average daily gain; lipopolysaccharides.

First, direct effects of the maternal treatments (Control, LPS, and β-glucan) on the performance of broiler breeders themselves were investigated (Table 2). Intratracheal treatment with LPS resulted in decreased egg production during the first week post inoculation (≈ 8%, *P* < 0.01; wk 34; Figure 2A and Table 2) compared to the control broiler breeder hens, but this reduced egg production was no longer significant one week later (wk 35). LPS treatment resulted in an increase in ADG in the second week after inoculation (∼4.1 g, *P* < 0.05; wk 34 to wk 35; Figure 2B and Table 2) compared to the control group. Effects on ADG were no longer present 1 wk later (wk 35 to 36). No differences on egg production and ADG were found for the β-glucan-treated hens. Furthermore, none of the maternal treatments showed an effect on body weight, ADFI, FCR, ME, egg weight, chick weight, YFBM, relative yolk weight of chick weight, Tona Score, fertility, mortality, and hatchability (Table 2 and Table S3: Egg and chick quality).

**Table 2:** Results of the broiler breeder phase (1). Effects of LPS and β-glucan treatment on BW, ADG, ADFI, FCR, Lay%, and ME of broiler breeder hens are shown. Measurements between 25 and 33 wk of age represents the pre-trial period data. Maternal treatment started at 33 wk of age (control, LPS, β-glucan). Broiler breeders were weighed individually weekly post treatment. The other parameters were measured and calculated during a time interval of a week. Egg collection for the grow-out phase was done during wk 35 and 36. Unless stated otherwise, results are shown as least squares means^1^.

Variable	Control	LPS	β-glucan	SEM	*P*-value	Average
Body weight (kg)						
Wk 33	3.797	3.778	3.799	0.055	0.542	3.791
Wk 34	3.824	3.803	3.831	0.058	0.464	3.819
Wk 35	3.849	3.857	3.871	0.052	0.687	3.859
Wk 36	3.883	3.882	3.899	0.054	0.735	3.888
Wk 25–33 Pre-trial period						
ADG. g	11.723	11.504	11.668	0.943	0.905	11.632
ADFI. g	155.2	154.2	155.5	0.8	0.354	155
FCR	3.669	3.625	3.742	0.107	0.175	3.679
Lay%	75.99	76.92	75.84	1.81	0.626	76.25
ME	42.45	42.64	41.83	1.26	0.435	42.31
Wk 33–34 Start LPS and β-glucan						
ADG. g	3.756	3.632	4.438	0.931	0.747	3.942
ADFI. g	161.8	160.8	161.3	0.6	0.525	161.3
FCR	3.003	3.103	3.005	0.053	0.322	3.037
Lay%	88.52^a^	83.73^b^	87.75^a^	1.09	0.007	86.66
ME	53.93	51.91	53.76	0.85	0.207	53.2
Wk 34–35						
ADG. g	3.628^a^	7.762^b^	5.825^ab^	1.259	0.037	5.739
ADFI. g	161.8	160.8	161.3	0.6	0.525	161.3
FCR	3.050	3.149	3.070	0.070	0.588	3.090
Lay%	87.13	82.56	86.13	1.6	0.125	85.27
ME	53.08	51.18	52.79	1.13	0.457	52.35
Wk 35–36 Egg collection for grow-out						
ADG. g	4.790	3.579	4.005	1.059	0.720	4.124
ADFI. g	161.8	160.7	161.3	0.6	0.440	161.3
FCR	2.939	2.963	2.989	0.051	0.792	2.964
Lay%	87.33	86.52	86.12	1.54	0.856	86.66
ME	55.07	54.35	54.06	0.91	0.731	54.49

Abbreviations: ADFI, average daily feed intake; ADG, average daily gain; BW, body weight; FCR, feed conversion ratio; Lay%, laying percentage; LPS, lipopolysaccharides.

1 Each treatment consisted of 6 pens with 27 broiler breeder hens per pen.

a-b Effects were considered to be significant when *P* ≤ 0.05.

**Figure 3:**
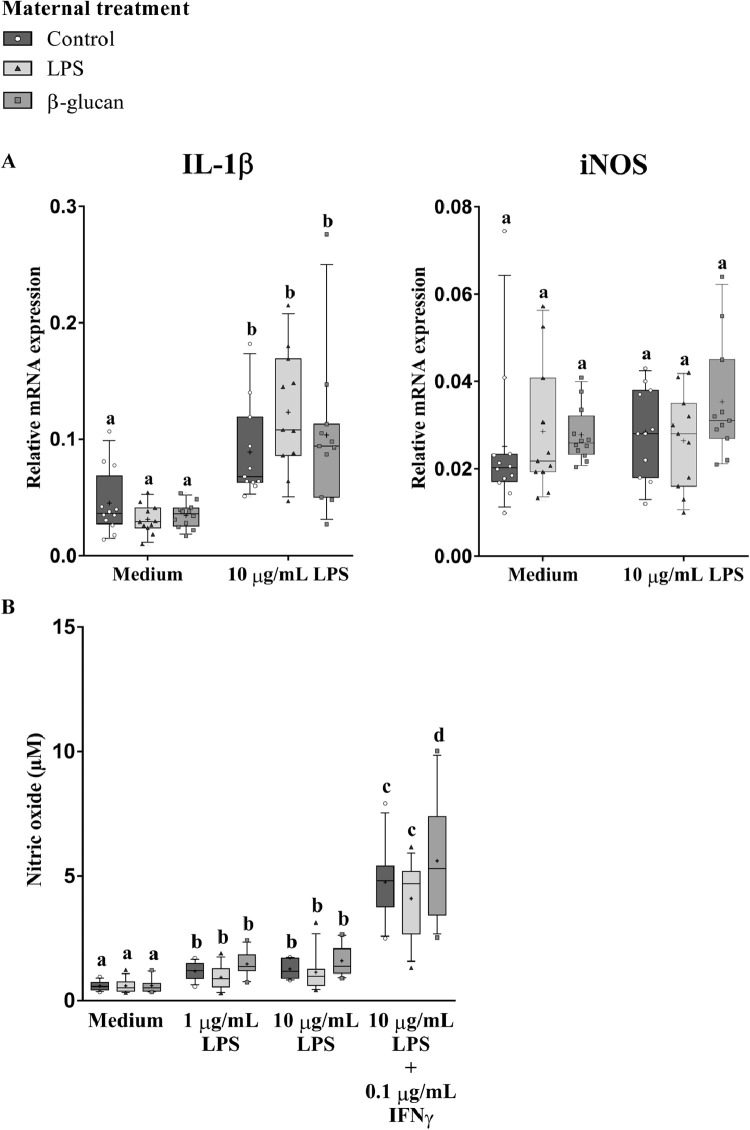
Effect of maternal treatments on inflammation associated parameters in blood-derived primary monocytes of broiler breeders. The broiler breeders received the maternal treatments at 33 wk of age and monocytes were collected at the age of 36 wk. (A) Relative mRNA expression levels of IL-1β and iNOS were measured after a 24 h ex vivo stimulation with medium or LPS (10 µg/mL), followed by collection of total RNA. (B) NO production levels in culture medium were measured after a 48 h ex vivo stimulation with medium or LPS (1 µg/mL, 10 µg/mL) or a combination of LPS (10 µg/mL) + IFNy (0.1 µg/mL). (A and B) N = 12 animals per treatment. For all figures the box extends from the 25th to 75th percentiles. The line in the middle of the box is plotted at the median and ‘+’ at the mean. Whiskers represents 10 to 90 percentile. Effects are represented as superscripts and were considered to be significant when *P* ≤ 0.05.

Next, effects of the in vivo maternal treatments (Control, LPS, and β-glucan) on ex vivo responsiveness of monocytes in the blood were investigated. Blood-derived monocytes were stimulated ex vivo and the mRNA expression of the inflammation-associated genes IL-1β and iNOS, and the production of NO were measured (Figure 3). Ex vivo stimulation with LPS resulted in increased IL-1β mRNA compared to ex vivo stimulation with only culture medium (*P* < 0.01), while the amount of iNOS mRNA remained unaffected (Figure 3A and Table S4). Furthermore, after the ex vivo LPS stimulation, no differences between the in vivo maternal treatments with either LPS or β-glucan has been found on IL-1β and iNOS expression levels compared to the control treatment (Figure 3A and Table S4).

Next, we measured the amount of NO accumulated in the culture medium upon agonist stimulation (Figure 3B). Irrespective of the source (control, LPS, or β-glucan treatment) of the monocytes, NO production increased after ex vivo stimulation with LPS (1 µg/mL, 10 µg/mL) or a combination of LPS (10 µg/mL) + IFNy (0.1 µg/mL) compared to the culture medium (control) group, whereby LPS + IFNy showed the largest NO production (*P* < 0.01; Figure 3B and Table S5). Interestingly, monocytes isolated from β-glucan-treated broilers displayed a significant (*P* < 0.05) higher NO production in response to ex vivo stimulation with LPS + IFNy, compared to monocytes from control or LPS-treated hens (Figure 3B and Table S5).

### Transgenerational Effect of Maternal Immunization on Growth Performance and Immune Responsiveness

**Figure 4:**
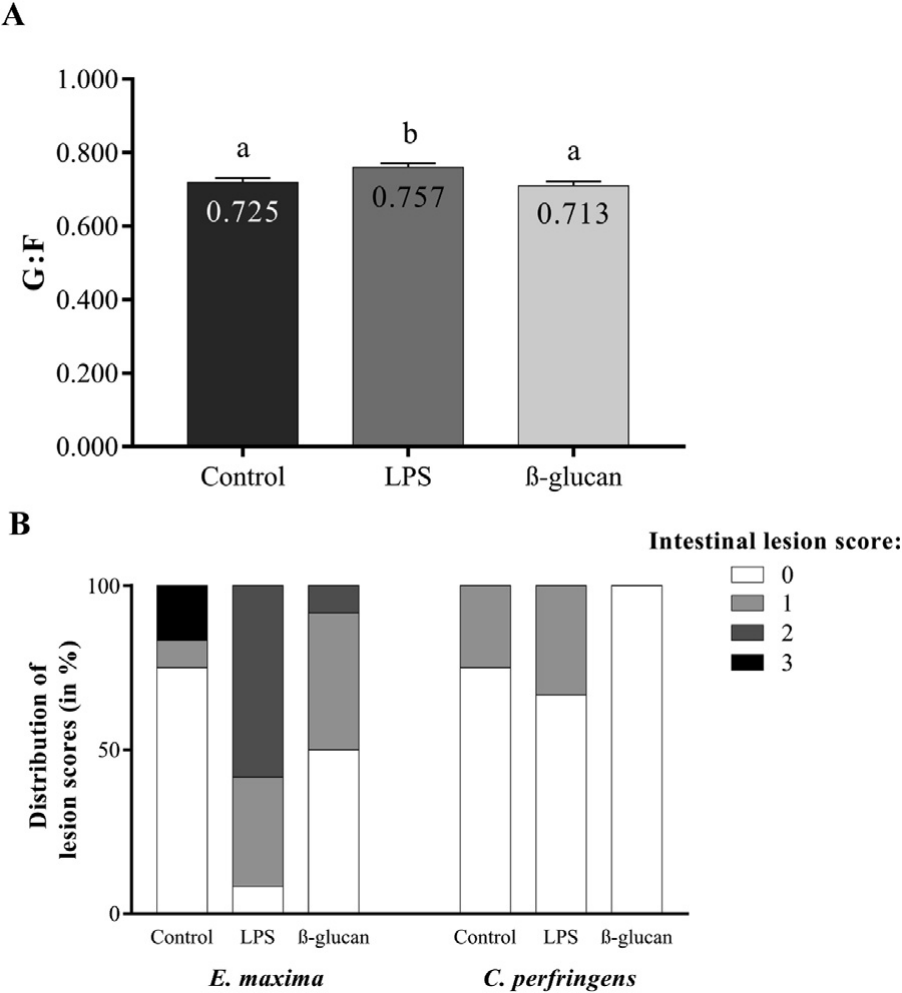
Effects of maternal treatment in the next generation on gain-feed ratio (G:F), *E. maxima* lesion scores and *C. perfringens* lesion scores, after the Necrotic enteritis (NE) challenge. The NE challenge consists of a *E. maxima* challenge at 7 days of age and a *C. perfringens* challenge at 14 days of age. A) Representation of the G:F from d 14 to d 21. Offspring related to the LPS-treated mother hens become more efficient in using feed after the NE challenge compared to the control group. N = 6 pens per treatment with 15 hens per pen. Data is represented as lsmeans. Effects are represented as superscripts and were considered to be significant when *P* ≤ 0.05. (B) Representation of the Necrotic enteritis lesion scores on d 15, 1 d after the *C. perfringens* challenge and 8 d after the *E. maxima* challenge. Data is represented as percentage of the total number of animals per treatment group. The distinction between the severeness of the intestinal lesions was also included in the figure (not included in statistical analysis). Zero indicates no lesions and 3 indicates the most severe intestinal lesions. More chicks showed *E. maxima* lesions that are related to the LPS treated broiler breeders compared to the control (*P* < 0.05). However, no significant effect of the *C. perfringens* infection was found with respect to the intestinal lesions. N = 6 pens per treatment with 2 hens per pen.

The offspring of the LPS-treated broiler breeders showed an increased gain feed ratio (G:F) during d 14 to d 21 compared to their associates of control and β-glucan-treated broiler breeders (*P* < 0.05; Table 3 and Figure 4A). NE intestinal lesions were measured on d 15, which was 1 d after the *C. perfringens* challenge and 8 days after the *E. maxima* challenge (Figure 4B). More broiler chickens with *E. maxima* lesion scores higher than 0 were found in the LPS treated broiler breeder offspring than in the control and β-glucan offspring (*P* < 0.05 with intestinal lesion score > 0; Figure 4B and Table S6). Furthermore, numeric differences were found between the severeness of the intestinal lesions, as visualised by the scores (0,1,2,3) (Figure 4B and Table S6). No effect of treatment was observed of *C. perfringens* infection on the intestinal lesion scores.

**Table 3:** Results of the grow-out phase (3) whereby the transgenerational effects of the maternal treatments (control, LPS, β-glucan) are investigated. Effects of LPS and β-glucan treatment on BW, ADG, ADFI, and G:F of the offspring before and after the NE challenge (d 15) are shown. On d 0 group BW was determined and averaged per pen. Individual BW were determined on d 7, d 14, d 21 and d 35. The other parameters were measured and calculated during a time interval of 7 d. Unless stated otherwise, results are shown as least squares means^1^.

Variable	Control	LPS	β-glucan	SEM	*P*-value	Average
Body weight (g)						
D 0	45.1	45.2	45.4	0.5	0.878	45.3
D 7	169.4	170.2	167.0	3.4	0.713	168.9
D 14	420.2	413.0	418.3	12.4	0.816	417.2
D 21	860.2	847.1	855.0	23.9	0.889	854.1
D 35	2,334.8	2,300.6	2,340.9	57.2	0.832	2,325.4
D 0 to 7						
ADG. g	17.75	17.86	17.22	0.451	0.533	17.61
ADFI. g	17.4	17.4	17.1	0.4	0.694	17.3
G:F	1.017	1.027	1.008	0.015	0.601	1.017
D 7 to 14						
ADG. g	35.83	34.68	35.91	1.60	0.612	35.47
ADFI. g	49.1	49.2	49.4	1.7	0.981	49.3
G:F	0.730	0.705	0.724	0.016	0.229	0.720
D 14 to 21						
ADG. g	62.41	61.98	61.78	2.01	0.971	62.06
ADFI. g	86.2	82.0	86.6	2.8	0.403	84.9
G:F	0.725^a^	0.757^b^	0.713^a^	0.011	0.017	0.731
D 21 to 35						
ADG. g	104.55	101.39	105.69	2.62	0.492	103.88
ADFI. g	154	150.5	156.7	4.0	0.462	153.7
G:F	0.679	0.674	0.675	0.009	0.888	0.68
D 0 to 35						
ADG. g	60.56	58.93	60.81	1.34	0.558	60.10
ADFI. g	85.2	82.8	86.3	2.0	0.454	84.8
G:F	0.711	0.712	0.705	0.005	0.367	0.71

Abbreviations: ADFI, average daily feed intake; ADG, average daily gain; BW, body weight; G:F, gain and feed ratio; LPS, lipopolysaccharides; NE, Necrotic enteritis.

1 Each treatment consisted of 6 pens with 15 male chickens per pen.

a-b Effects were considered to be significant when *P* ≤ 0.05.

**Figure 5:**
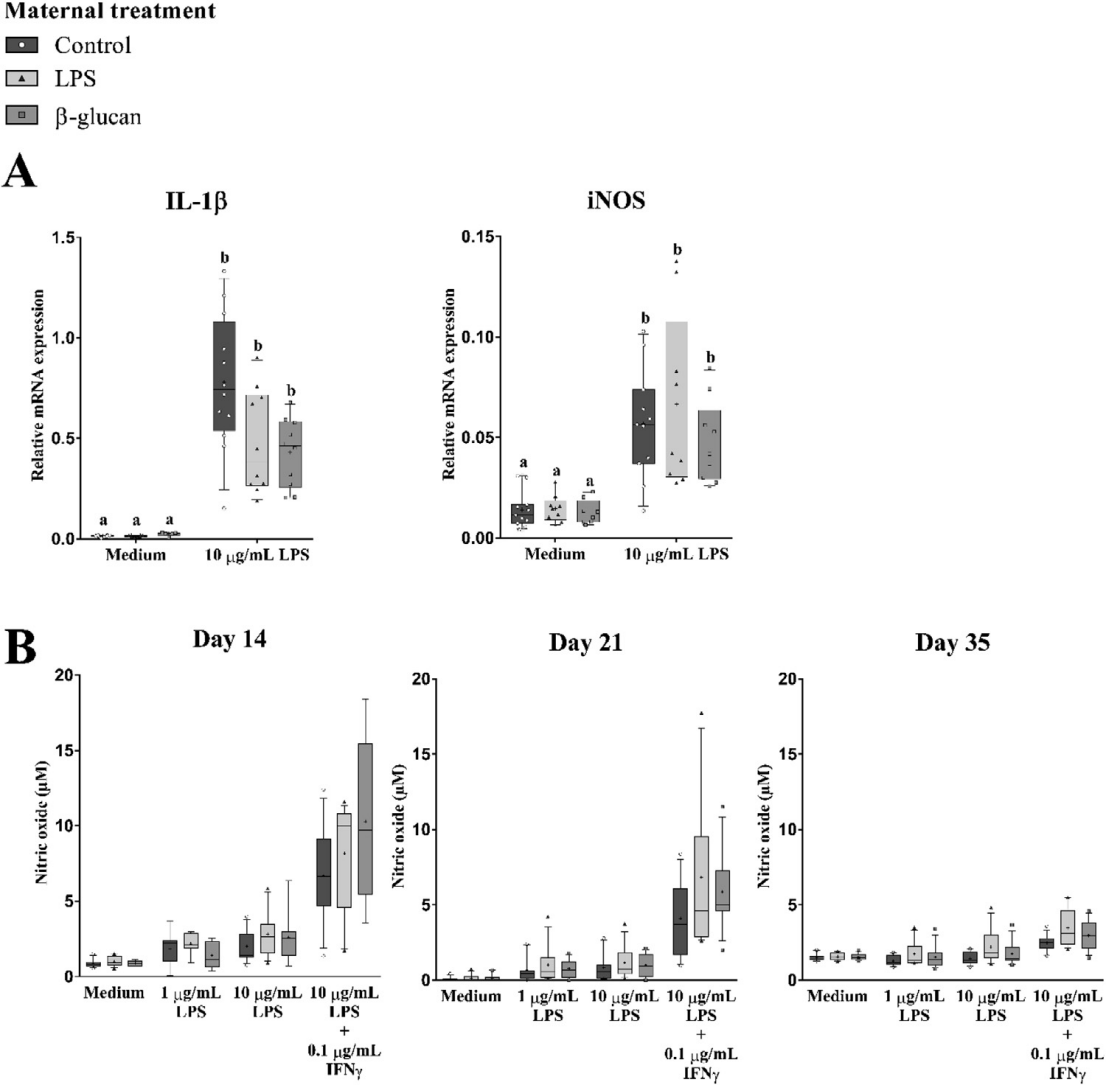
Effects of maternal treatment on the responsiveness of blood-derived primary monocytes isolated in the next generation (grow-out phase 3). Gene expression levels and NO production were measured after ex vivo stimulation with medium or LPS. Offspring received in vivo at d 14 the *C. perfringens* infection. (A) Relative mRNA expression levels of IL-1β and iNOS were measured in the monocytes that were collected at the age of 21 d (7 d after the in vivo challenge). Monocytes received ex vivo stimulation with medium or LPS (10 µg/mL) for 24 h. (B) Nitric oxide production of primary monocytes collected at d 14, d 21 and d 35. Monocytes were ex vivo stimulated with medium or LPS (1 µg/mL, 10 µg/mL) or a combination of LPS (10 µg/mL) + IFNy (0.1 µg/mL) for 48 h. (A and B) N = 12 animals per treatment. The box extends from the 25th to 75th percentiles. The line in the middle of the box is plotted at the median and ‘+’ at the mean. Whiskers represents 10 to 90 percentile. Effects are represented as superscripts and were considered to be significant when *P* ≤ 0.05. Abbreviations: iNOS, inducible nitric oxide synthetase; LPS, lipopolysaccharides; NO, nitric oxide.

Ex vivo LPS stimulation of blood-derived monocytes from 21-day-old offspring resulted in increased IL-1β and iNOS mRNA levels compared to the culture medium-treated group (*P* < 0.01; Figure 5A and Table S7). Furthermore, both in vivo maternal treatment LPS and β-glucan tended (*P* = 0.08) to decrease IL-1β mRNA in monocytes exposed to ex vivo LPS stimulation (Figure 5A and Table S7). The LPS-induced iNOS mRNA expression, on the contrary, was not affected by the maternal treatments.

Notably, NO production in response to LPS decreased in cells derived from 35-day-old broiler chickens compared to those from 21 or 14 days old. (*P* < 0.01). Monocytes from chickens at 14 days of age showed a higher (*P* < 0.01) LPS-induced NO production compared to those from chickens of 21 or 35 days of age (Figure 5B and Table 4). Furthermore, NO production was higher in ex vivo LPS or LPS+IFNy-stimulated cells compared to the culture medium group (*P* < 0.01, Figure 5B and Table 4). Highest NO production was observed in cells stimulated with LPS+IFNy. NO levels were higher in the in vivo maternal LPS treatment group compared to the control group (*P* < 0.05; Figure 5B and Table 4). No difference between the β-glucan maternal treatment group was found compared to the control (Figure 5B and Table 4)

**Table 4:** Statistical output grow-out phase (3): Nitric oxide production. Accumulated NO in the medium upon ex vivo LPS stimulation in the grow-out phase (3) was determined and used as a tool to investigate transgenerational effects of the maternal treatments (control, LPS, β-glucan).

Variable	Category	n	LSMeans	SEM	*P*-value
Maternal treatment	β-glucan	141	2.45^a^	0.17	0.0141
	LPS	136	2.73^b^	0.17	
	Control	131	2.02^a^	0.17	
Age	14	124	3.36^a^	0.18	0.0077
	21	144	1.88^b^	0.16	
	35	140	1.96^b^	0.17	
Ex vivo LPS stimulation	0	104	0.86^a^	0.19	<0.0001
	1	96	1.45^ab^	0.20	
	10	104	1.72^b^	0.19	
	10+0.1 IFNy	104	5.57^c^	0.19	
R-square model		0,52			

Interaction age × Ex vivo LPS stimulation *P* < 0.01.

a,b,c Different letters *P* < 0.05.

#### Transgenerational Effect on Agglutinating Antibody Titers

**Figure 6:**
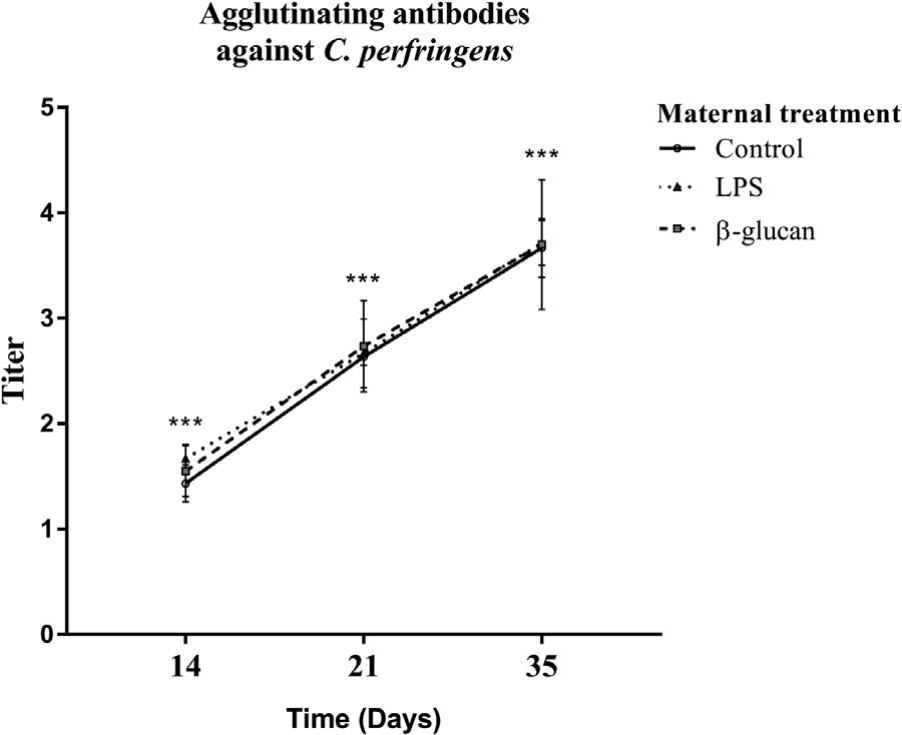
Agglutinating antibody titers against *C. perfringens* in the offspring's blood plasma. Offspring received an oral inoculation of *C. perfringens* at d 14. Blood plasma was collected at d 14 (before infection), d 21 and d 35 of age and subjected to an agglutination assay. Each bar represents the mean of agglutinating antibody titers ± SEM with N = 30 animals per treatment. * *P* < 0.05; ** *P* < 0.01; *** *P* < 0.001.

We measured agglutinating antibody titers as a serologic marker to investigate if maternal treatments would influence the humoral response in the offspring after a challenge with *C. perfringens*. Antibody titers against *C. perfringens* increased in all chickens of all ages. The antibody titers increased with age (*P* < 0.01), but were not affected by the in vivo maternal treatments (Figure 6).

## DISCUSSION

In this study, we aimed to investigate if maternal stimulation of the innate immune system with MAMPs in broiler breeders could have an effect on growth performance parameters and immune responsiveness of their offspring. Both MAMP treatments of the broiler breeder phase are extensively used in research on trained innate immunity in mammals (Quintin et al., 2012; Novakovic et al., 2016). These researchers found epigenetic reprogramming of trained monocytes, indicating a role for innate training in disease protection at the longer term. β-glucan has been found to induce trained innate immunity in mammals and chickens, which results in an increased immune response after a second stimulation with an unrelated antigen (Bekkering et al., 2016; Verwoolde et al., 2020a). Within the broiler breeder phase, dietary treatment with β-glucan did result in increased NO production levels of monocytes, suggesting innate immune activation. However, no further effects of β-glucan treatment on growth performance and immune responses were observed. LPS stimulation has also been found to initiate functional reprogramming of innate immune cells, including monocytes in mammals (Novakovic et al., 2016). In contrast to the β-glucan treatment, the LPS treatment reduced egg production and increased growth directly after administration. Intratracheal challenges with LPS has pathological consequences for chickens, whereas oral administration of beta-glucans has not (Wideman et al., 2004; Ploegaert et al., 2007; Cox and Dalloul, 2010). These pathological consequences are due to the proinflammatory effects of LPS (Cheng et al., 2004). Maternal LPS treatment may therefore result in stronger transgenerational effects compared to the β-glucan treatment. Indeed, the transgenerational effects in the present study are specifically found in the offspring of LPS-treated broiler breeder hens.

The greater G:F ratio in the offspring of LPS-treated broiler breeder hens during the first week following the *C. perfringens* challenge, could be ascribed to a transgenerational effect on feed conversion efficiency. It is possible that the retainment of nutrients recovers more quickly in broiler chickens from the maternal LPS treatment group despite the higher occurrence of intestinal lesions. Since the G:F was measured during 7-d intervals and intestinal lesion score only at d 15, it is tempting to speculate that the intestinal tract of the offspring of the LPS-treated broiler breeders had an improved and faster recovery and therefore a greater G:F ratio, while the recovery of the control group was slower. It would be interesting to investigate whether the recovery rate of the capacity of nutrient absorption in the offspring is enhanced by maternal LPS treatment of broilers in a future study. The observation of the intestinal lesions caused by *E. maxima* could be an indication of transgenerational innate training effects due to maternal immunization with LPS. For future research it is recommended to measure lesion scores at shorter intervals after challenge to visualize the dynamics of the appearance of intestinal lesions in time and consequently see intestinal recovery in time. Intestinal recovery from a bacterial challenge may be an evaluation parameter to explore transgenerational effects.

The tendency for a treatment effect of maternal immunization with MAMP on IL-1β mRNA expression by ex vivo LPS stimulated monocytes of offspring needs to be confirmed. Furthermore, it is unclear whether reduced IL-1β transcription levels also result in reduced IL-1β protein levels and as a consequence reduced inflammation. Whether this reduced IL-1β mRNA expression is caused by epigenetic modifications in monocytes or by attenuated infection due to increased resistance is currently not know.

We applied β-glucan through the feed, since it is a commonly used immune modulating feed additive (Volman et al., 2008; Morales-López et al., 2009; Chou et al., 2017). We used LPS intratracheally, because this infection model was already successfully used in the past to stimulate the immune system of chickens (Parmentier et al., 2008; Parmentier et al., 2010). Considering the results of LPS and β-glucan in the offspring of the current study, it is conceivable that a more pathological treatment, such as LPS, has the potential to initiate a more detectable transgenerational effect compared to a more mild treatment such as dietary β-glucan. Differences in animal age, β-glucan dosage, exposure time, β-glucan purity, and β-glucan origin might be key factors in inducing transgenerational effects (Zhang et al., 2005; Owens and McCracken, 2007; Cox and Dalloul, 2010; Tang et al., 2011; Cho et al., 2013).

We found low mortality rates and relatively low intestinal lesion scores upon the NE challenge in the offspring, indicating that we applied a subclinical infection. Subclinical conditions can be preferable to study immune-related effects. In contrast to overstimulation of the immune system induced by a severe challenge, a mild infection may increase the chance of finding more subtle differences in immune responses. Furthermore, subclinical conditions represent the most frequent situation under practical circumstances. The infection model used in this study, is therefore highly suitable for testing the effect of feed induced improvements of animal health.

Within the current study, it was also hypothesized that differences in monocyte activity would influence the agglutinating capability against *C. perfringens*. The cytokine IL-1β is an activator of the humoral response and would therefore also influence the production of antibodies against *C. perfringens* (Nakae et al., 2001; Ritvo and Klatzmann, 2019). However, despite the fact that we did observe decreased mRNA levels of IL1β after the *C. perfringens* infection in the LPS treatment group, no differences in the level of specific antibodies was found.

The importance of transgenerational effects has not been recognized for a long time, but it now becomes more clear that these effects have long lasting effects on the physiology of an organism (Gluckman et al., 2007; Godfrey et al., 2007; Berghof et al., 2013). In this study, we aimed to investigate if activation of the maternal innate immune system with MAMPs affects performance and innate immune responsiveness of the offspring. These data are a first indication that broiler breeder hens can affect immune responsiveness and feeding efficiency of their offspring in a transgenerational manner. Studies with pied flycatchers and domesticated zebra finches describe transgenerational effects of maternal immunization with LPS on antibody levels in the offspring (Grindstaff et al., 2006; Grindstaff et al., 2012; Merrill and Grindstaff, 2014). However, no mechanism behind this transgenerational effect has been described. It is possible that modification of the innate immune system by molecules such as LPS is responsible for the described effects. For future studies, we would recommend additional MAMPs that are known to be able to train the innate immune system (Rusek et al., 2018) in combination with additional read-out parameters. More read-out parameters which have already described to be associated in visualizing trained innate immunity and transgenerational epigenetics in mammals, including histone modification analyses or transcriptomics, may be worth considering (Quintin et al., 2012; Perez and Lehner, 2019). In addition, it should be realized that vaccinations of breeder hens could also have transgenerational effects on offspring immune phenotype (Hasselquist and Nilsson, 2009). Administration of standard vaccines to broiler breeders could therefore be an useful strategy to study transgenerational effects. More knowledge about transgenerational effects of maternal immunization or infection will contribute to a better understanding of the variation in immune phenotypes, disease resistance and metabolic disorders.

## Appendix Supplementary materials

Image, application 1

## Footnotes

Supplementary material associated with this article can be found, in the online version, at doi:10.1016/j.psj.2021.101413.
