# Supplementary material for: Transgenerational effects of innate immune activation in broiler breeders on growth performance and immune responsiveness
Source: Poult Sci. 2021 Jul 30;100(11):101413. doi: 10.1016/j.psj.2021.101413 (PMC8531860; doi:10.1016/j.psj.2021.101413)
Supplement: Supplementary file 1 [file mmc1.pdf]

SUPPLEMENTARY MATERIAL

Materials & Methods

Tables & Figures

Table S1

Table S1. Ingredient and nutritional composition of the starter, grower and finisher diets during the Grow-out phase.

	Starter	Grower	Finisher
Soybean meal	305.1	250.6	246.6
Maize	285.9	356.4	352.7
Wheat	200.0	200.0	200.0
Soybeans, heat treated	80.0	80.0	80.0
Rapeseed, solvent extracted	50.0	50.0	50.0
Animal fat,	20.1	16.1	20.5
Soybean oil	20.1	16.1	20.5
Limestone	14.76	11.78	11.81
Monocalciumphosphate	6.69	2.51	2.58
Sodiumbicarbonate	2.6	2.66	1.99
Salt	1.9	1.93	1.88
DL-Methionine	2.14	1.53	1.29
L-Lysine HCL	0.51	0.37	-
L-Threonine	0.11	0.00	-
Hostazym X 15000	0.1	0.1	0.1
Phyzyme XP 10000 TPT - 500 FTU	0.05	0.05	0.05
Premix Broiler Starter[1]	10	-	-
Premix Broiler Grower[2]	-	10	10
CP	243	221	219
Crude fat	77	71	79
Ash	0	0	0
DM	888	884	885
ME[3], kcal/kg	2850	2900	2950
Dig. Lys[3]	12.0	10.6	10.2
Ca	8.8	6.8	6.8
P	5.7	4.6	4.6
CP	247	227	225
Crude fat	80	73	94
DM	885	881	882

Ca	8.2	6.5	6.7
P	5.2	4.3	4.6

[1]Supplied per kg diet: thiamine, 1.0 mg; riboflavin, 4.5 mg; niacinamide, 40 mg; D-pantothenic acid, 9 mg; pyridoxine-HCL, 2.7 mg; choline chloride, 500 mg; cyanocobalamin, 20 µg; vitamin E (DL-α-tocopherol), 33 IU; menadione, 2.3 mg; vitamin A (retinyl-acetate), 12,000 IU; cholecalciferol, 5,000 IU; biotin, 100 µg; folic acid, 0.5 mg; $FeSO_4 \cdot H_2O$, 150 mg; MnO_2, 100 mg; $CuSO_4 \cdot 5H_2O$, 40 mg; $ZnSo_4 \cdot H_2O$, 145 mg; Na_2SeO_3, 0.56 mg; KI, 2.0 mg; antioxidant (oxytrap PXN), 125 mg.

[2]Supplied per kg diet: vitamin A (retinyl-acetate), 10,000 IU; vitamin D3 (cholecalciferol), 2,000 IU; vitamin E (DL-α-tocopherol), 20 mg; vitamin K3 (menadione), 2.3 mg; vitamin B1 (thiamine), 0.8 mg; vitamin B2 (riboflavin), 4.5 mg; vitamin B6 (pyridoxine-HCL), 1.9 mg; vitamin B12 (cyanocobalamine), 20 µg; niacine, 30 mg; D-pantothenic acid, 8 mg; choline chloride, 400 mg; folic acid, 0.5 mg; biotin, 50 µg; $FeSO_4.H_2O$, 150 mg; $CuSO_4.5H_2O$, 40 mg; MnO, 100 mg; $ZnSo_4.H_2O$, 145 mg; KI, 1.9 mg; Na_2SeO_3, 0.50 mg; antioxidant (oxytrap PXN), 125 mg

[3] calculated according to CVB (2018).

Table S2

Table S2. Ingredient (g/kg) and nutritional composition of experimental diets during the broiler breeder phase.

	Control and LPS	β-glucan
Maize	405.0	404.5
Wheat	200.0	200.0
Soybean meal	129.7	129.7
Wheat middlings	100.0	100.0
Sunflowerseed meal	50.0	50.0
Soybean oil	22.3	22.3
Limestone	76.3	76.3
Sodiumbicarbonate	2.57	2.57
Salt	2.27	2.27
Monocalciumphosphate	0.40	0.40
DL-Methionine	1.19	1.19
L-Threonine	0.17	0.17
Hostazym X 15000	0.10	0.10
Phyzyme XP 10000 TPT - 500 FTU	0.05	0.05
Premix[1]	10.0	10.0
β-glucan (MacroGard)	-	0.50
CP	157.0	157.0
Crude fat	44.0	44.0
Ash	105.0	105.0
DM	889.0	889.0
ME[2], kcal/kg	2850.0	2850.0
Dig. Lys[2]	5.7	5.7
Ca	30.0	30.0
P	4.2	4.2
CP	157.4	157.4
Crude fat	44.4	44.4
DM	889.4	889.4
Ca	30.0	30.0
P	4.2	4.2

[1]Supplied per kg diet: Vitamin A (retinyl-acetate), 12,500 IU; vitamin D3 (cholecalciferol), 2,500 IU; vitamin E (DL-α-tocopherol), 80 mg; vitamin K3 (menadione), 2.5 mg; vitamin B1 (thiamine), 2.0 mg; vitamin B2 (riboflavin), 9.0 mg; vitamin B6 (pyridoxine-HCL), 4.5 mg; vitamin B12 (cyanocobalamine), 30 µg; niacine, 40 mg; D-pantothenic acid, 12 mg; choline chloride, 600 mg; folic acid, 1.5 mg; biotin, 150 µg; FeSO4.H2O, 135 mg; CuSO4.5H2O, 40 mg; MnO, 130 mg; ZnSo4.H2O, 206 mg; KJ, 2.6 mg; Na2SeO3, 0.9 mg.

[2] calculated according to CVB (2018).

Materials & Methods

Animals, Housing and Management

Broiler breeder phase (1)

Diets were formulated based on digestibility and nutrient data provided by CVB (CVB. 2018b) in Bestmix (version 3.30.93). Diet composition was based on broiler breeder guide recommendations (version 2017, Aviagen-EPI, Roermond, The Netherlands) for the starter phase up to 5% lay and for the peak phase (5% lay to end of the trial). Feed was produced in mash form by Research Diet Services (Wijk bij Duurstede, the Netherlands). Target body weights were the same for all broiler breeders and followed breed recommendations (version 2017, Aviagen-EPI, Roermond, The Netherlands).

Egg incubation phase (2)

The eggs were stored at 20°C when stored for 3 days or 15°C when stored up to 6 days, room humidity was maintained between 70 and 80%. After 6 days, eggs were incubated at 37.5°C and 55% humidity using two incubators (NatureForm Inc., Jacksonville, FL). The 15 eggs per pen were distributed over 18 trays, with in each tray 6 blocks (collection day) with 15 eggs each. Broiler breeder pens were randomly allocated within blocks. After the setter period (d0 to d17 of incubation), eggs were transferred to baskets, based on the blocking and tray structure in the setter. From d19 to d21 of incubation, temperature was decreased to 36.7°C and 70.0% humidity.

Grow-out phase (3)

All treatments received the same diets, which were formulated based on digestibility and nutrient data provided by CVB (CVB. 2018b) in Bestmix (version 3.30.93). Diet composition was based on CVB recommendations for broilers: maximum growth (CVB. 2018a) and fed in three phases: starter (d0 to d7), grower (d7 to d21) and finisher (d21 to 35) (**Table S1**). All

diets were produced in pelleted form (2.5 mm starter and grower, 3.0 mm finisher) by Research Diet Services (the Netherlands).

Performance Data Collection

Egg incubation phase (2)

Chick quality was assessed using part of the score as described by Tona et al. (2003). Activity was scored 0 (good) or 6 (weak), down and appearance was scored 10 (clean and dry), 8 (wet) or 0 (dirty and wet) and the navel was scored 12 (closed and clean), 6 (not completely closed but not discolored) or 0 (not closed and discolored). The total score was calculated by adding the individual scores per chick. Subsequently, the same chicks were weighed, killed by cervical dislocation and dissected to measure residual yolk weight and calculate yolk free body mass (YFBM). Yolk weight as percentage of live body weight was calculated.

Immunological Data Collection

Alamar blue assay

Alamar blue (AB) is a non-toxic cell-permeable solution and was added to the cells directly after the collection of the culture media for the NO assay. Stock solution was made by dissolving 1 g of Resazurin Sodium Salt (BioReagent, R7017, Sigma-Aldrich Chemie GmbH, Schnelldorf, Germany) in 100 mL sterile PBS (Gibco) followed by sterile filtering through a 0.22 µm pore size syringe filter (Acrodisc, Pall Laboratory Corporation, New York, NY, USA) and stored at -20°C protected from light until further use. A 250x dilution in pre-warmed culture medium was used for the analysis with a total end volume of 100 µl per well. Cells with AB solution were incubated at 41°C in 5% CO_2 and 95% humidity for 4 hours protected from light. Reduction was quantified by measuring the optical density at 570 nm and 600 nm with a spectrophotometer at 0 h and 4 h (Multiscan™, Thermo Fisher Scientific, Waltham, MA, USA). Percentage of reduced AB solution for both time point was calculated

using the following formula, based on AB assay manual (Biosource version PI-DAL1025/1100Rev 1.0, Invitrogen, Carlsbad, California, USA):

$$percentage\ reduced = \frac{(\varepsilon_{OX})\lambda_2 A\lambda_1 - (\varepsilon_{OX})\lambda_1 A\lambda_2}{(\varepsilon_{RED})\lambda_1 A'\lambda_2 - (\varepsilon_{RED})\lambda_2 A'\lambda_1}$$

λ_1 = 570 nm λ_2 = 600 nm
(ε_{OX}) λ_2 = molar extinction coefficient of AlamarBlue oxidized form 600 nm
(ε_{OX}) λ_1 = molar extinction coefficient of AlamarBlue oxidized form 570 nm
(ε_{RED}) λ_2 = molar extinction coefficient of AlamarBlue reduced form 600 nm
(ε_{RED}) λ_2 = molar extinction coefficient of AlamarBlue reduced form 570 nm
A λ_2 = absorbance of test wells 600 nm
A λ_1 = absorbance of test wells 570 nm
A λ_2' = absorbance of negative control well 600 nm
A λ_1' = absorbance of negative control well 570 nm

Results

Table S3

Table S3. Effects of maternal treatment on egg and chick quality.

Variable	Control	LPS	β-glucan	SEM	P-Value	Average
Egg Weight Start, g	62.45	62.10	62.66	0.38	0.53	62.41
Chick Weight d0, g	45.22	45.34	45.41	0.40	0.93	45.32
YFBM, %	39.54	39.52	39.71	0.34	0.87	39.59
Relative Yolk Weight, %	12.35	12.50	11.66	0.36	0.18	12.17
Tona Score [1]	17.49	17.28	17.40	15.17	0.55	17.39
Fertility [2]	0.981	0.972	0.983	0.007	0.401	0.979
Mortality [2]	0.043	0.030	0.045	0.009	0.415	0.040
Hatchability [2]	0.932	0.921	0.924	0.012	0.763	0.926

[1] *Box-Cox transformation was applied to indicated variables owing to their non-normal distribution. Reported LSMeans and Average have been back-transformed for ease of interpretation*

[2]*Probability of a lower score*

Table S4

Table S4. Broiler breeder phase (1): Relative mRNA expression (Age=week 36)

		Gene iNOS				Gene il1β			
Variable	Category	n	LSMeans	SEM	P-value	n	LSMeans	SEM	P-value
Treatment	Control	23	0.027	0.003	0.4055	23	0.07	0.01	0.6999
	LPS	22	0.028	0.003		22	0.08	0.01	
	β glucan	23	0.031	0.003		23	0.07	0.01	
Ex vivo LPS stimulation	0	35	0.027	0.002	0.3275	35	0.04	0.01	<0.0001
	10	33	0.030	0.002		33	0.11	0.01	
R-square model		0.04				0.43			

Table S5

Table S5. Broiler breeder phase (1): Nitric oxide production (Age=week 36)

Variable	Category	n	LSMeans	SEM	P-value
Treatment	Control	48	1.94^{b}	0.15	0.0117
	LPS	48	1.68^{b}	0.15	
	β glucan	48	2.31^{a}	0.15	
Ex vivo LPS stimulation	0	36	0.59^{a}	0.12	<0.0001
	1	36	1.19^{b}	0.12	
	10	36	1.33^{b}	0.12	
	10+0.1 IFNy	36	4.82^{c}	0.12	
R-square model		0.74			

a,b,c: different letters P < 0.05

Table S6

Table S6. Grow-out phase (3): NE challenge lesion scores in percentage (Age=day 15)

E. maxima				*C. perfringens*			
	treatment				treatment		
Lesion score	Control	LPS	β-glucan	Lesion score	Control	LPS	β-glucan
0	75.00	8.33	50.00	0	75.00	66.67	100.00
1+2+3	25.00[a]	91.67[b]	50.00[a]	1+2+3	25.00[a]	33.33[a]	0.00[a]
**Total**	**100.0**	**100.0**	**100.0**	**Total**	**100.0**	**100.0**	**100.0**
0	75.00	8.33	50.00	0	75.00	66.67	100.00
1	8.33	33.33	41.67	1	25.00	33.33	0.00
2	0.00	58.33	8.33	2	0.00	0.00	0.00
3	16.67	0.00	0.00	3	0.00	0.00	0.00
**Total**	**100.0**	**100.0**	**100.0**	**Total**	**100.0**	**100.0**	**100.0**

Table S7

Table S7. Grow-out phase (3): Relative mRNA expression (Age=day 21)

		Gene iNOS				Gene il1B			
Variable	Category	n	LSMeans	SEM	P-value	n	LSMeans	SEM	P-value
Treatment	Control	23	0.044	0.009		20	0.41	0.06	0.0842
	LPS	19	0.038	0.010		18	0.24	0.06	
	β-glucan	19	0.040	0.010		16	0.26	0.07	
Ex vivo LPS stimulation	0	28	0.014	0.008	<0.0001	21	0.01	0.06	<0.0001
	10	33	0.068	0.008		33	0.60	0.05	
R-square model		0.30				0.59			

References

CVB. 2018a. Tabellenboek veevoeding pluimvee.
CVB. 2018b. Veevoedertabel.
